# Sugarcane apoplast fluid modulates the global transcriptional profile of the diazotrophic bacteria *Paraburkholderia tropica* strain Ppe8

**DOI:** 10.1371/journal.pone.0207863

**Published:** 2018-12-14

**Authors:** Paula Renata Alves da Silva, Márcia Soares Vidal, Cleiton de Paula Soares, Valéria Polese, Michelle Zibetti Tadra-Sfeir, Emanuel Maltempi de Souza, Jean Luiz Simões-Araújo, José Ivo Baldani

**Affiliations:** 1 Department of Crop Science—UFRRJ, BR 465, Seropédica–RJ–CEP, Brazil; 2 Embrapa Agrobiologia, Seropédica–RJ–CEP, Brazil; 3 Departament of Biochemistry and Molecular Biology, Centro Politecnico—UFPR, Rua XV de Novembro, Curitiba–PR–CEP, Brazil; Estacion Experimental del Zaidin, SPAIN

## Abstract

The stalk apoplast fluid of sugarcane contains different sugars, organic acids and amino acids that may supply the demand for carbohydrates by endophytic bacteria including diazotrophs *P*. *tropica* (syn. *B*. *tropica*) strain Ppe8, isolated from sugarcane, is part of the bacterial consortium recommended as inoculant to sugarcane. However, little information has been accumulated regarding this plant-bacterium interaction considering that it colonizes internal sugarcane tissues. Here, we made use of the RNA-Seq transcriptomic analysis to study the influence of sugarcane stalk apoplast fluid on Ppe8 gene expression. The bacterium was grown in JMV liquid medium (100 ml), divided equally and then supplemented with 50 ml of fresh JMV medium or 50 ml of apoplast fluid extracted from sugarcane variety RB867515. Total RNA was extracted 2 hours later, the rRNAs were depleted and mRNAs used to construct libraries to sequence the fragments using Ion Torrent technology. The mapping and statistical analysis were carried out with CLC Genomics Workbench software. The RNA-seq data was validated by RT-qPCR using the reference genes *fliP1*, *paaF*, and *groL*. The data analysis showed that 544 genes were repressed and 153 genes were induced in the presence of apoplast fluid. Genes that induce plant defense responses, genes related to chemotaxis and movements were repressed in the presence of apoplast fluid, indicating that strain Ppe8 recognizes the apoplast fluid as a plant component. The expression of genes involved in bacterial metabolism was regulated (up and down), suggesting that the metabolism of strain Ppe8 is modulated by the apoplast fluid. These results suggest that Ppe8 alters its gene expression pattern in the presence of apoplast fluid mainly in order to use compounds present in the fluid as well as to avoid the induction of plant defense mechanisms. This is a pioneer study showing the role played by the sugarcane apoplast fluid on the global modulation of genes in *P*. *tropica* strain Ppe8.

## Introduction

The apoplast is defined as a highly dynamic compartment which connects roots to leaves in a continuum flow through the stems and it has been considered a very important channel related to the perception and transduction of signals from the environment to the symplast (intracellular) [1]. It has been suggested that diazotrophs prefer the plant apoplast fluid since the symplast restricts ions, sugar and other solutes due mainly to the permeability of the membrane [2]. The presence of the endophytic diazotrophic bacteria *Gluconacetobacter diazotrophicus* living in the fluid of sugarcane apoplast was demonstrated [3] and later its presence was confirmed within the xylem and apoplast fluid of sugarcane [4]. Isolates of *Azospirillum* have been found within surface disinfected roots of sugarcane, therefore suggesting that it endophytically colonizes sugarcane [5]. *Herbaspirillum seropedicae* has also been found colonizing the apoplast tissue of sugarcane leaves very close to vascular parenchyma cells [6]. It has been suggested that apoplast fluid is one of the most suitable niches for baterial endophytes [7, 8]. In addition to reducing the competition, the apoplast fluid can supply various organic and inorganic compounds.

The apoplast fluid of sugarcane plants contains sugars, amino acids, proteins, ammonium nitrate and nitrite which can change according to the variety and type of plant management [9, 10]. Aconitic acid, malate and citrate have been found among the organic acids while sucrose, glucose and fructose were among the sugars detected. Indeed, aconitic acid and sucrose predominate among the organic acids and sugars present in various different varieties and also at different phenological stages [9]. These organic acids and sugars are probably essential for endophytic diazotrophic bacteria as they exchange the fixed nitrogen in any portion of the stem and at different sugarcane plant ages [9].

The genus *Burkholderia* contains 96 species that comprise one of the most versatile groups of pathogenic and nonpathogenic bacterial species living in water, soil and/or associated with plants [11]. This genus was recently divided and the genus *Paraburkholderia* was created [12, 13]. The new genus comprehends 46 species isolated mainly from soil, rhizosphere soil and plant tissues and includes the older nitrogen–fixing species *B*. *tropica*, *B*. *unamae* and *B*. *silvatlantica*, all associated with non-legume plants [14–16]. *Paraburkholderia tropica* Ppe8 strain was isolated from the stems of sugarcane plants grown in the Northeast of Brazil and is part of the sugarcane inoculant consortium developed by Embrapa [15]. This strain presents optimum growth between pH 5.0–5.8, a pH in the range of that reported (5.5) for the apoplast fluid of sugarcane [3]. It has been reported that *P*. *tropica* uses various carbon sources including, sugars and organic acids as energy sources [15]. In addition to the positive inoculation response observed when it is present in the sugarcane inoculant consortium [17], it has also been reported colonizing endophytically tomato plants and promoting an increase in fruit yields [18] as well as showing potential for biological control of *Colletotrichum gloesporioides*, *Sclerotum rolffsi*, *Fusarium culmorum* and *F*. *oxysporum* fungi both *in vitro* and *in vivo* [19]. In addition to fixing nitrogen, this strain produces siderophore [15, 20], solubilizes inorganic P and produces indols [20].

Despite all these traits indicating the strain as a good biofertilizer candidate, little information has been accumulated regarding its interaction with the plant, in particular the sugarcane host. In contrast, results indicating that other *Paraburkholderia* species may recognize the plant and respond through the induction or repression of genes have already been published [21, 22]. The transcriptomic study of *P*. *phytofirmans* strain PsJN inoculated in two potato varieties showed that about 62% of the genes were expressed during the interaction [22]. In this study, it was verified that most of the differentially expressed genes were related to transcriptional regulation, general metabolism (sugars, amino acids, lipids and nucleotides), energy production and cellular homeostasis [22]. In addition, it was found that genes related to motility and defense mechanisms seem to be less important for the successful establishment of the endophytic bacteria. Another transcriptomic study involving the *P*. *kururiensis* strain M130 cultivated in the presence of rice plant extract showed that genes involved in flagella biosynthesis were mostly downregulated, suggesting a mechanism to allow the bacteria to escape host plant defense responses [21].

Considering the biotechnological importance of the bacteria to the sugarcane crop and the scarcity of information accumulated in relation to its interaction with plants, this study aims to explore the role of stalk sugarcane apoplast fluid on the gene expression of *P*. *tropica* strain Ppe8, expecting to identify bacterial genes regulated by the apoplast fluid compounds during *in vitro* interaction.

## Materials and methods

### Harvesting of stalk apoplast fluid

The apoplast fluid was collected from 10 month old (mature plants) sugarcane variety RB867515 according to the methodology described by [3]. The plants were grown in an Argisol type soil at the field experimental station of Embrapa Agrobiologia, located in Seropédica (latitude -22°44’49.33” and longitude -43°40’15.75”). The entire stems were harvested and immediately transported to the laboratory. They were washed with tap water and soap and then disinfected with alcohol 70%. The disinfected stems were peeled and the internode was cut into sections approximately 5 cm in length (proportional to the falcon tube size), followed by a new disinfection with alcohol 70% and flaming for the evaporation of symplast fluid. A support was placed on the basal extremity to avoid the contact of apoplast fluid with the stem during centrifugation. The falcon tubes were maintained on ice to reduce the oxidation of the samples until centrifugation at 3,000 *x* g for 20 minutes at 4°C. The apoplast fluid samples were mixed and filtered in Millipore filtres (0.22 μm) to remove microganisms, then transferred to a new sterile falcon, and stored in the utrafreezer at -70°C. Each replicate was supplemented the same apoplast fluid and consequently the organic/chemical compounds were equal.

### Bacterial growth

*Paraburkholderia tropica* strain Ppe8, obtained from the Embrapa Agrobiologia Biological Resource Center (BRC), was grown in flasks containing 100 ml of defined JMV liquid medium [23] modified by replacing mannitol by sucrose as carbon source in same proportion. Sodium glutamate (10 mM) was used as N source. Once the cells reached the mid log phase (O.D._600nm_ ~ 0.3 and 10^8^ CFU/ml), the culture was divided into two equal portions (50 ml) and the following treatments were applied: a) addition of 50 ml of fresh modified JMV medium (MM) and b) addition of 50 ml of apoplast fluid of sugarcane variety RB867515 (MLA). Two hours later, 5 ml of cells were collected for total RNA extraction (centrifugation at 9,000 *x* g, 4°C for ten minutes). There were three biological replications for each treatment.

### Transcriptomic profile and analysis

The total RNA was isolated with Trizol in accordance with manufacturer protocol (Life Technologies) and treated with DNase I (Epicenter) for the removal of genomic DNA contamination. RNA purity was quantified using Qubit (Life Technologies). Seven micrograms of total RNA was used to ribosomal RNA (rRNA) depletion with MICROBExpress kit. The efficiency of depletion was evaluated in agarose gel electrophoresis (1%) followed by quantification of the total RNA with Agilent 2100 Bioanalyzer (Agilent). A total of 500 ng mRNA was used for the construction of a sequencing library using the standard protocol of the SOLid Total RNA-Seq Kit (Life Technologies). The libraries were barcoded by using the SOLiD Transcriptome Multiplexing Kit (Life Technologies). The emulsion PCR and sequencing were performed according to Ion One Touch 200 Template Kit v2 DL and Ion PI Sequencing 200 Kit v2 using the standard Life Technologies protocols, respectively. The Ion Proton Semiconductor Sequence (Life) was used to sequence six libraries generated from three biological replicates from each independent treatment.

The genome sequence data are available in the DDBJ/ENA/GenBank and published with the accession number MSDZ00000000.1; BioSample: SAMN06166932.

### Data analyses

Mapping the reads against the genome sequence of *P*. *tropica* strain Ppe8, data processing and statistical analysis were performed using the CLC Genomics Workbench (v. 6.5.1). The reads were first quality trimmed (quality score higher than 0.05 and reads with less than 20 bp were discarded) followed by mapping with the following parameters: 90% minimum alignment to Ppe8 reference sequence, 80% minimum identity and the number of hits equal 1 for inclusion as mapped read. RPKM values were generated using default parameters for CLC Genomics. Genes were considered differentially expressed when the fold change was higher than 2 or smaller than -2 and the p-value smaller than 0.05 by test of Baggerley’s corrected for false discovery rate (FDR). These differentially expressed genes were categorized through the WebMGA online software [24]. Pathways from COG database were used for this analysis and only differentially expressed genes with a FDR smaller than 0.05 were considered.

### Validation of the RNA-Seq transcriptome by RT-qPCR

For validation with Quantitative reverse transcription PCR (RT-qPCR), total RNA was isolated from cultures grown in presence and absence of apoplast fluid using the Trizol (Life Technologies) and quantified using Qubit (Life Technologies). The RNA was extracted as described above. The cDNAs were synthesized using the kit Superscript III Reverse Transcriptase (Invitrogen). The gene expression was quantified using the GoTaq qPCR Master Mix (Promega) on a Step One Plus Real Time-PCR System (Applied Biosystems). The Primer3 plus software [25] was used to design the primers. The *lpxC* and *recA* genes were used as reference genes for RT-qPCR [26], and the relative gene expression was determined using the qBase v.1.3.5 [27]. The Cq values of the genes evaluated in this study were obtained from the Miner software. The PCR reaction consisted of 7.5 μl of GoTaq qPCR Master Mix, different concentrations of forward and reverse primers were utilized (Table 1) as well as 5.0 μl of 1:20 diluted cDNA template in a total volume of 15 μl. Cycling was performed using the default conditions of the 7500 Software v 2.0.5: 2 min at 95°C, followed by 40 cycles of 20 s at 95°C and 30 s at different temperatures (Table 1). All RT-qPCR assays were carried out using three technical replications and non-template controls, as well as three independent cDNA syntheses. Correlation analysis between the relative expression of RT-qPCR and RNA-seq data of differentially expressed genes was performed (Minitab, v.15.0).

**Table 1:** Primers used for validation of RNA-seq transcriptome by RT-qPCR.

Gene	Primer ID	5'-3' sequence ()	Size of amplicon	Primer concentration	Anneling temperature ()
*groL*	groLF	GGCAACTACGGCTACAACG	174 bp	500 pmol	65°**C**
	groLR	CATCGGTGCATCTTCCTTC			
*fliP1*	fliP1F	CATTCCGTTCCTCATCATC	71 bp	500 pmol	62°**C**
	fliP1R	GCGAGACCATCATCATACC			
*paaF*	paaFF	TTACACCGCCAAGGACATC	106 bp	500 pmol	62°**C**
	paaFR	GCCATAGCCGTAACTCACG			
*lpxC*	lpxCF	TGAAGACGGTCGGCATCGGC	112 bp	100 pmol	65°**C**
	lpxCR	ACGGGGGTCGGCAAATCCAC			
*recA*	recAF	TCTGGACATTCGCCGTATCG	141 bp	500 pmol	60°**C**
	recAR	GAGATGCCTTCGCCGTAGAG			

## Results

### Changes in the transcriptome of *P*. *tropica* strain Ppe8 in response to sugarcane apoplast fluid

The transcriptional profile of *P*. *tropica* strain Ppe8 in presence and absence of sugarcane apoplast fluid generated approximately 17 million reads for both treatments, with approximately 1.7 million mapped only to the genome of strain Ppe8 when cultured in the presence of 50% liquid apoplast (MLA) and and 2.1 million mapped in the presence of 50% of fresh modified JMV medium (MM), respectively (Table 2).

**Table 2:** Data from the RNA-seq analysis for *P*. *tropica* strain PPe8 grown in presence and absence of apoplast fluid from sugarcane variety RB867515.

Samples	Number of reads for each biological replicate	Total of reads	Number of reads after removal of rRNAs	Number of reads mapped exclusively in the genome of the strain Ppe8/replicate	Total of reads mapped exclusively in each treatment
MLA R1	4,698,029	16,786,088	1,942,888	346,311	1,765,132
MLA R2	7,806,802		4,467,387	920,308	
MLA R3	4,281,257		1,633,248	498,513	
MM R1	3,767,829	16,236,734	1,710,185	629,250	2,158,245
MM R2	7,444,118		2,671,227	1,088,477	
MM R3	5,024,787		2,896,158	440,518	

The reads were mapped to the genome of *P*. *tropica* strain Ppe8 with the CLC Genomics Workbench v.5.1 software, with a minimum length of 90% and similarity of 80. The numbers R1, R2 and R3 refer to the replicates of the biological samples of MLA and MM, respectively.

**Fig 1:**
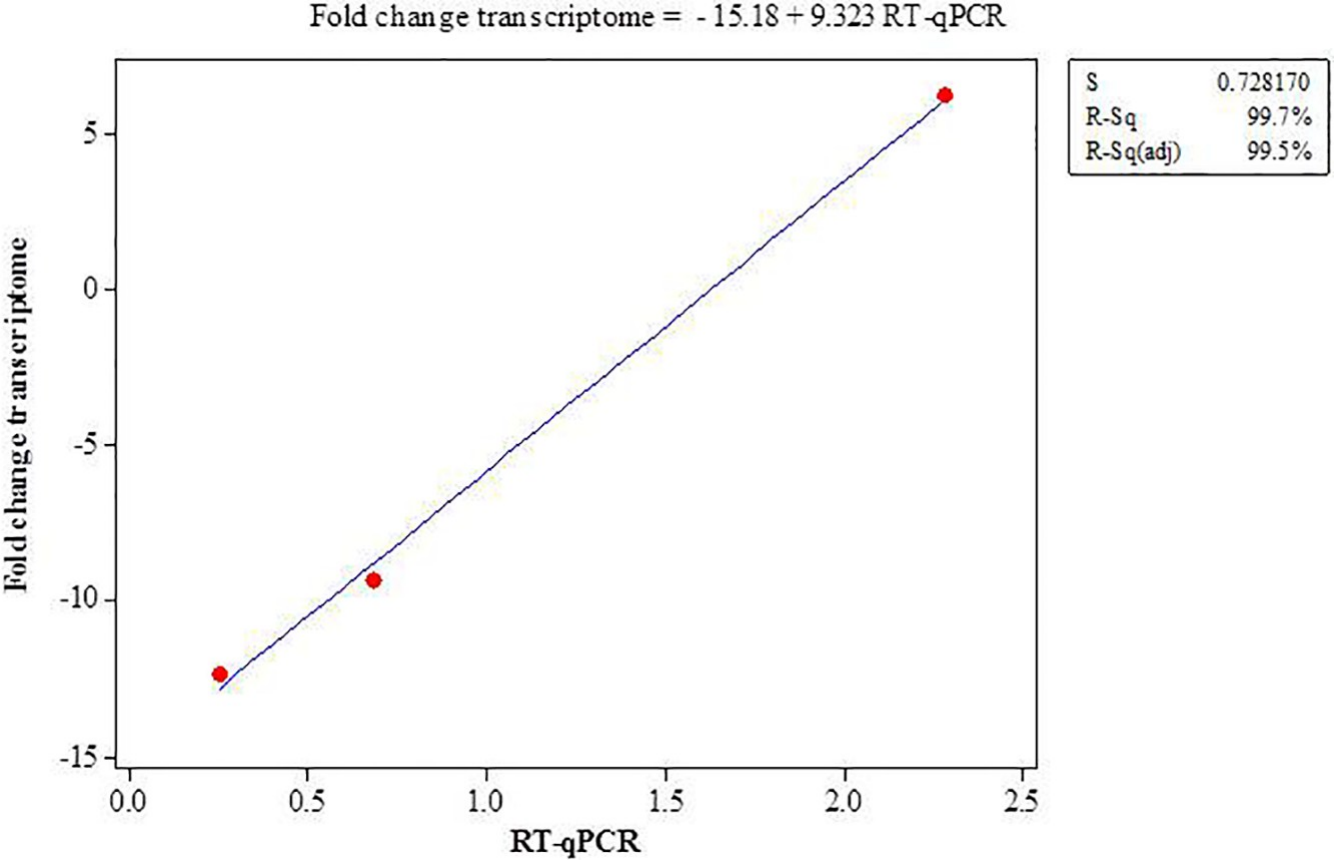
Correlation analysis between RNA-seq and RT-qPCR analysis of the differential genes expression in Ppe8 strain in MLA medium in comparasion with MM medium (calibrator = 1). The data of RT-qPCR were expressed in relative expression and the data of RNA-seq in fold change.

Data analysis carried out with the CLC Workbench software showed that 544 genes were repressed and 153 genes induced in the presence of apoplast fluid (MLA) compared to the defined medium (MM) (S1 Table). The data obtained by RNA-seq were then validated through the RT-qPCR technique using three random selected genes that were differentially expressed. The reference genes *lpxC* and *recA*, already defined for this bacterial species, were used as internal controls [26]. The expression pattern observed by RNA-seq analysis for genes *fliP1*, *paaF* and *groL* in strain Ppe8 cultured in presence of apoplast fluid was confirmed by RT-qPCR (Table 3). A high correlation (R^2^ = 99%) was observed between both techniques for all three genes analyzed (Fig 1).

**Table 3:** Genes differentially expressed in the *P*. *tropica* strain Ppe8, grown in MM (defined medium) (calibrator = 1) in comparison to MLA (apoplast fluid medium).

Gene	Fold change transcriptome	RT-qPCR^a^
*fliP1*	-12.4	0.25
*paaF*	-9.4	0.68
*groL*	6.2	2.28

^**a**^relative expression

**Fig 2:**
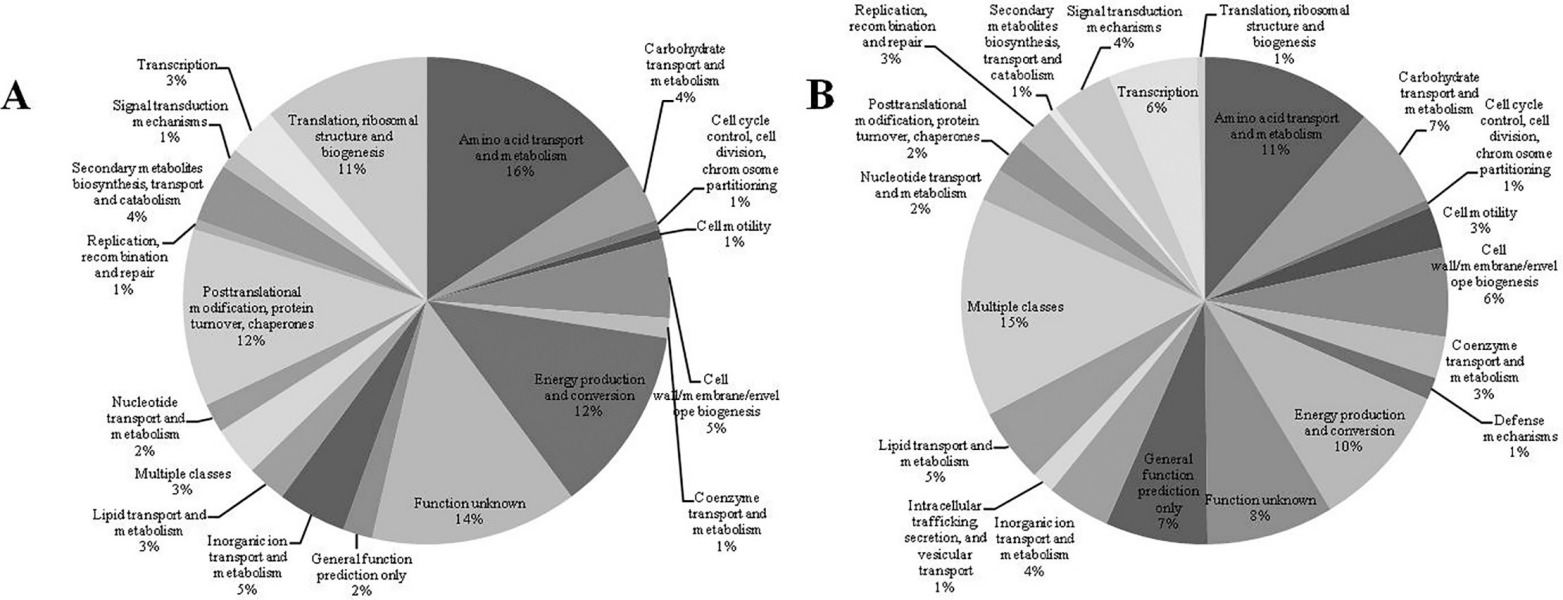
Functional classification of genes upregulated and downregulated in strain Ppe8 grown in presence of sugarcane apoplast fluid. One hundred fifty-three were upregulated (A) and 544 were downregulated (B) in presence of apoplast fluid. The genes were functionally classified by COG (Clusters of Orthologous Groups of Proteins) (http://www.ncbi.nlm.nih.gov/COG), [28].

The majority of induced genes belong to the functional classes of metabolism and amino acids transport, unknown function, posttranslational modification, protein and chaperone recovery and biogenesis, ribosomal structure and translation (Fig 2A). On the other hand, repressed genes belong to the multiple classes of metabolism and amino acid transport, energy production and conversion, unknown function and carbohydrate metabolism and transport (Fig 2B).

### Expression of cell wall-related genes are altered in strain Ppe8 grown in the presence of sugarcane apoplast fluid

There were differential expressions of cell wall-related genes when strain Ppe8 was grown in the presence of apoplast fluid as compared to defined medium. Eight genes were overexpressed while 32 genes were repressed for functional classes of cell all, membrane and envelope biogenesis. Among the repressed genes were found the *ompA* genes coding for porin (outer membrane protein), and the ORFs *_1009*, *ORF_1011* and *ORF_2630*, which encode the biosynthesis of exopolysaccharide. Cultivation in the presence of apoplast fluid also repressed genes encoding glycosyltransferases (*ORF_986*, *ORF_999*, *ORF_1005*, *ORF_1008*, *ORF_991* and *ORF_866*), among others. A gene encoding a mannose-1-phosphate guanyltransferase (*cpsB*) and belonging to the same class was repressed in the presence of apoplast fluid (S1 Table). Genes encoding glycosyltransferases were also repressed in the presence of apoplast fluid (S1 Table).

### Expression of cell motility and chemotaxis related genes are affected in strain Ppe8 cultivated in the presence of apoplast fluid

**Fig 3:**
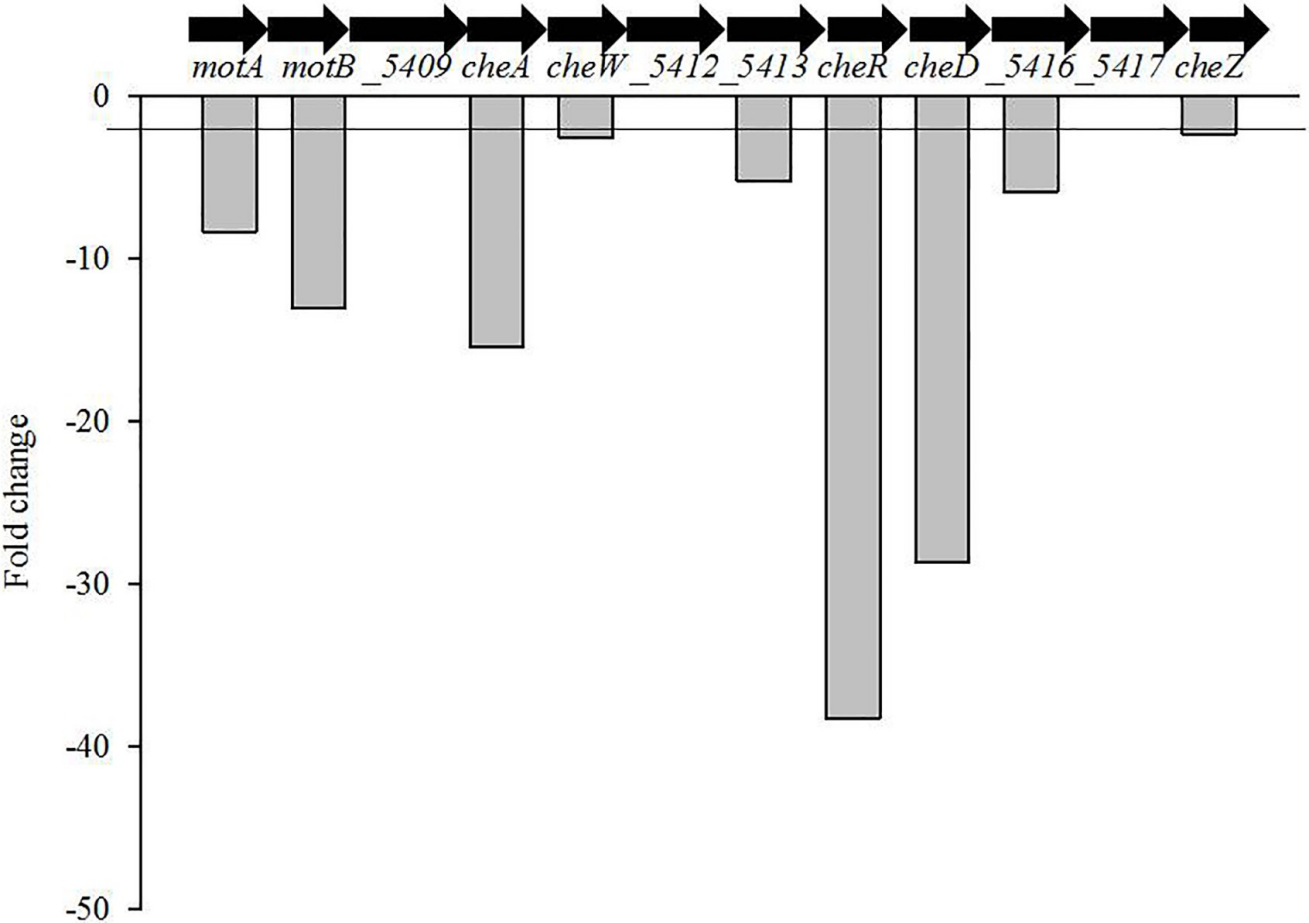
Genes of the cell motility and chemotaxis are repressed in strain Ppe8 grown in the presence of apoplast fluid in comparasion with defined medium. Genes without bars (Fold change) were not differentially expressed. The genes with fold change equal or minor that -2 were considered repressed in strain Ppe8 in presence of sugarcane apoplast fluid.

**Fig 4:**
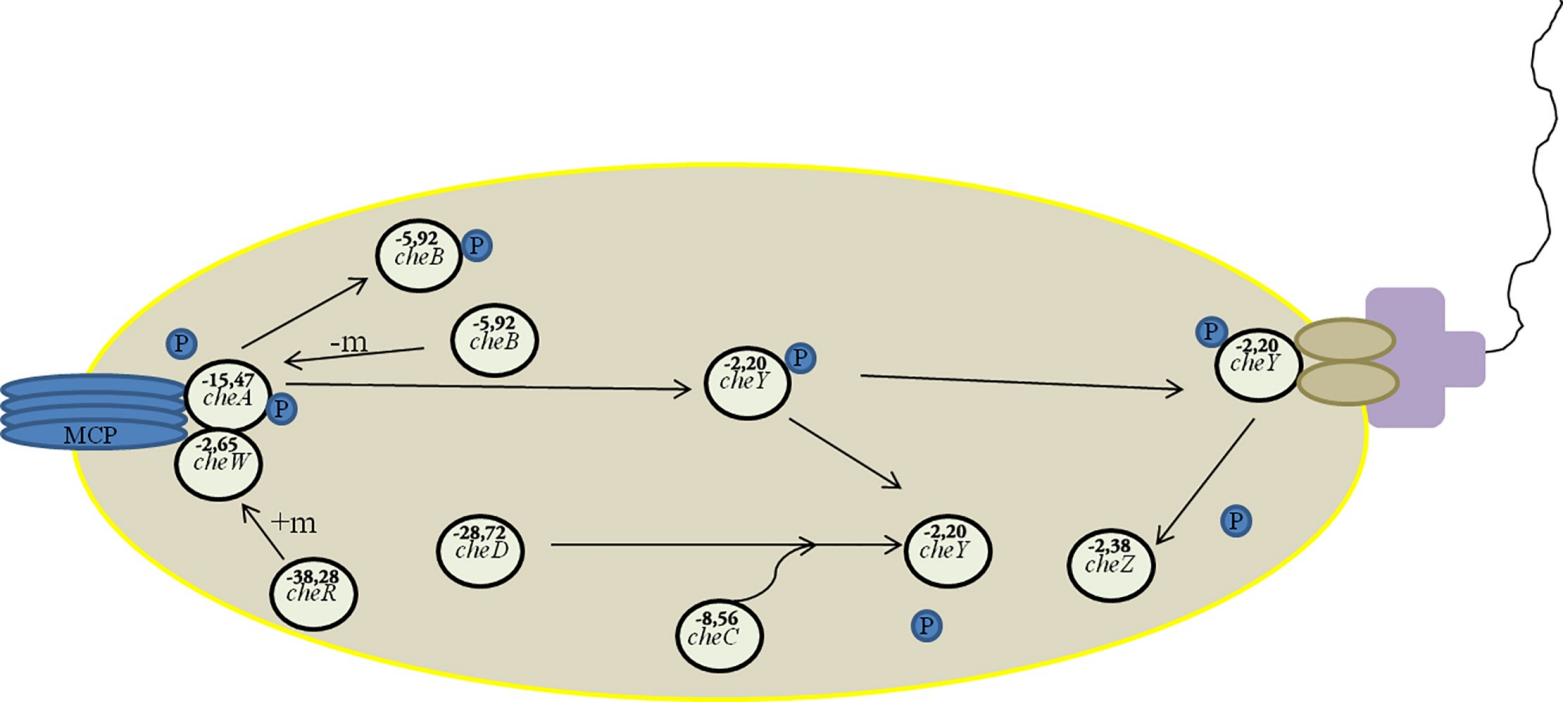
Regulatory cascade of chemotaxis (Source: Adapted from Russell, 2012 [29]). The genes depicted in the figures that participate of chemotaxis in Ppe8 strain, were repressed in the presence of apoplast fluid in comparasion with defined medium. The genes with fold change equal or minor that -2 were considered repressed in strain Ppe8 in presence of sugarcane apoplast fluid.

Few genes involved with chemotaxis and cell motility were repressed in presence of sugarcane apoplast fluid. They are: *cheA*, *cheC*, *cheD cheW*, *cheZ*, *cheY* and *cheR* (conserved group of a signal transduction system) that encode chemotaxis-related proteins (Fig 3) and ORFs: *_1925*, *_3609*, *_5847*, *_6798* and *_7470* (known as MCPs or methyl-accepting proteins), a group of transmembrane chemoreceptors (Fig 4).

**Fig 5:**
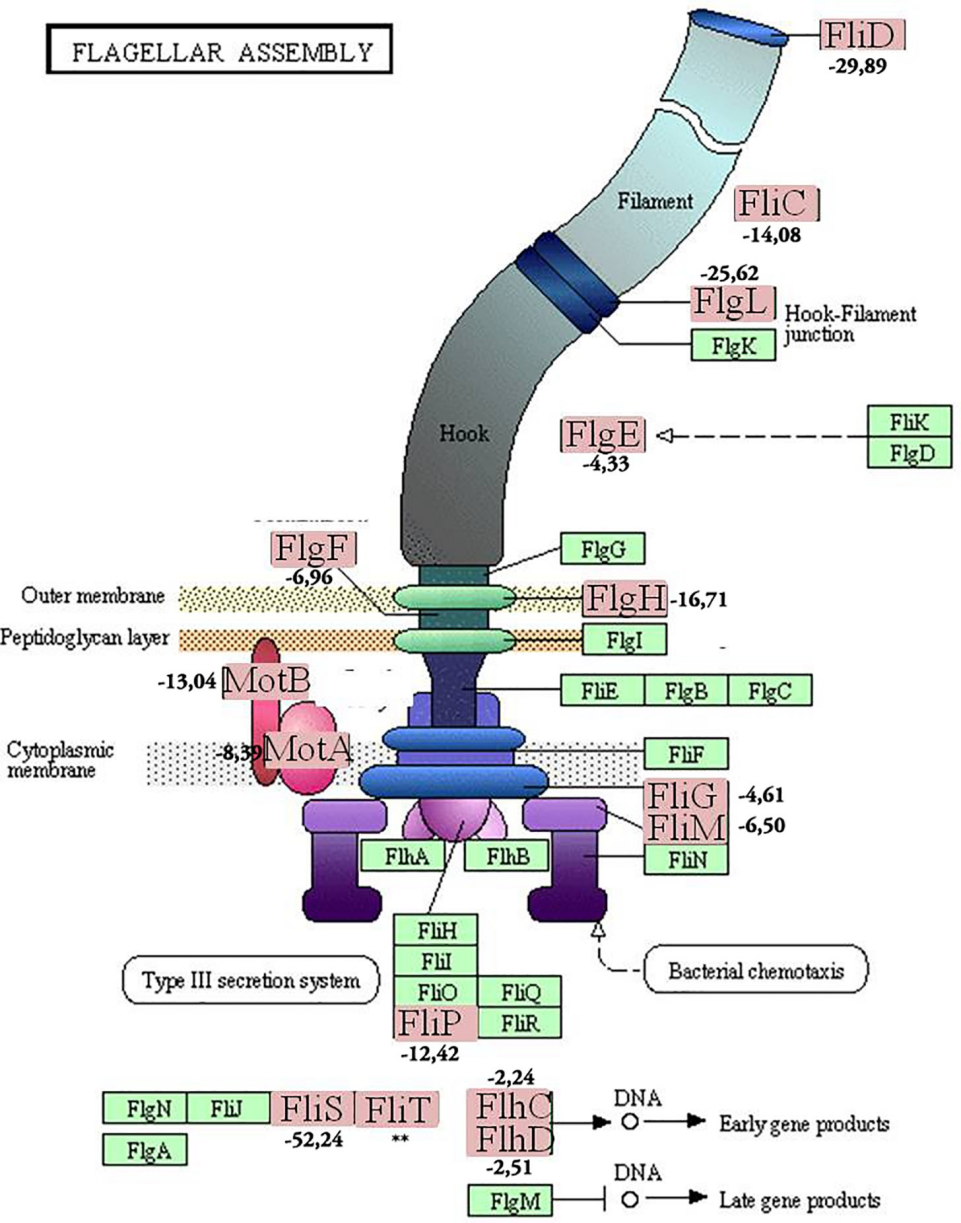
Proteins involved in flagellar assembly (Source: Adapted from KEGG[30]). The set of genes present in genome of *P*. *phytofirmans* is represented in the green box. This genome is one of the closest, phylogenetically, to plant endophytic *Paraburkholderia* species that have been published. Genes that are in the light red boxes were repressed in strain Ppe8 in the presence of apoplast fluid in comparation with defined medium. The genes with fold change equal or minor that -2 were considered repressed in strain Ppe8 in presence of sugarcane apoplast fluid. Reprinted from Kanehisa et al. under a CC BY license, with permission from KEGG, original copyright [2016].

The genes responsible for the flagella *flgF*, *flgH*, *fliM*, *fliT*, *flhD*, *flhC*, *fliP1*, *fliD1*, *flgE*, *motA*, *motB*, *fliG*, *flgL*, *fliC* and *fLiS* were also repressed in the presence of apoplast compounds (Fig 5) (S1 Table).

### Catabolism of secondary metabolites and transport

The genes, *pcaG* and *pcaH*, involved in protocatechol catabolism; *paaB*, phenylacetic acid degradation protein; *ORF_4074*, 4-hydroxyphenylpyruvate dioxygenase; *ORF-36*98, Anthraniloyl-CoA monooxygenase, and the *kdsA1* gene, 4-Hydroxy-2-oxoglutarate aldolase, were found overexpressed when the strain was cultured in the presence of apoplast fluid. In contrast, the ORFs, *_1634* and *_7508* encoding dienelactone hydrolase and 2-keto-3-deoxy-L-fuconate dehydrogenase, respectively and the *phbC* gene encoding a poly-beta-hydroxybutyrate polymerase were repressed (S1 Table).

### Signal transduction mechanisms

Twenty-two genes that participate in the pathway of signal transdution mechanisms were repressed in strain Ppe8 cultured in the presence of apoplast fluid whereas only two genes were induced. Among the repressed genes were histidine kinases (*ORF_7341*, *ORF_3321*, *ORF_5560*, and *ORF_926*), responsive regulators (*ORF_1924* and *ORF_3322*), GGDEF, EAL, GAF or PAS/PAC domain proteins (*ORF_4086*, *ORF_1999*, *ORF_4017*, *ORF_4100*, *ORF_6195*, *ORF_1916* and *ORF_5976*), among others (S1 Table). The two induced genes are *acpD* and *ompR* that encode FMN-dependent NADH-azoreductase and two-component system response regulator, respectively (S1 Table).

### Metabolism and transport of amino acids and carbohydrates

Among the functional classes of metabolism and transport of amino acids and carbohydrates there were 32 genes induced and 99 genes repressed in *P*. *tropica* strain PPe8 growth in the presence of apoplast fluid. These results suggest that the apoplast fluid modulates the metabolism of the bacteria according to the presence of compounds (S1 Table).

Several genes belonging to classes of carbohydrate metabolism and transport such as the *gntP* and *edd* genes (gluconate transporter family protein and phosphogluconate dehydratase) and the ORFs: _*2932*, _*3932*, _*5824* and _*6424* (glycerol-3-phosphate ABC transporter, glyoxalase family protein, N-ethylmaleimide reductase and aldehyde dehydrogenase, respectively) were induced in the presence of the apoplast fluid compared to defined medium. However, there was a strong repression of genes belonging to this class with 37 genes repressed in the presence of apoplast, 12 of which belonged to ABC type sugar carriers (S1 Table).

Another interesting aspect was that many more genes related to carbon and transport systems were repressed in strain Ppe8 cultivated in apoplast liquid than in the defined medium. They were *xylF*, the ORF encoding L-arabinose-binding periplasmic protein (_*1244*), fucose operon *fucU* (_*2355*), ribose ABC transport system (_*2359*, _*2360* and _*6379*), xylose isomerase (_*801* and _*6322*) and monosaccharide ABC transporter (_*820* and _*1904*) (S1 Table).

The presence of apoplast fluid changes the expression pattern to several genes classified in the metabolism and transport of amino acid classes in strain Ppe8. The number of repressed genes was higher than the induced ones, 62 genes were repressed and 26 genes induced under the same growth conditions (S1 Table). Among the induced genes, *glt1* (glutamate synthase), *hutH* (histidine ammonia lyase), *aroE* (shikimate 5-dehydrogenase), *argC* (acetylglutamate kinase), *aspS* (aspartate-tRNA ligase), *opucA* (N-acetyl-gamma-glutamyl-phosphate reductase), *gcvP* (glycine dehydrogenase), *gcvT* (glycine cleavage of the T protein system), among others, were all observed (S1 Table). In contrast, the apoplast fluid also repressed genes. Out of the 62 repressed genes, 12 are ABC type transporters (S1 Table).

## Discussion

### Changes in the transcriptome of *P*. *tropica* strain Ppe8 in response to sugarcane apoplast fluid

A total of 697 genes were differentially expressed in the genome of *P*. *tropica* Ppe8 strain when the CLC Workbench software was applied (S1 Table), (8.89% approximately) suggesting that the apoplast fluid modulates the gene expression in strain Ppe8 when the bacteria are present in the inner tissues of sugarcane plants. The literature has already shown that apoplast fluid changes the gene expression of *Pseudomonas syringae*, *Ralstonia solanacearum*, and *Xanthomonas campestris* associated with beans, tomato and cabbage, respectively [31–33].

### Expression of genes involved the cell wall are altered in strain Ppe8 grown in presence of apoplast fluid

The gene *ompA* coding for porin and the ORFs *_1009*, *_1011* and *_2630*, which encode exopolysaccharide biosynthesis and those encoding glycosyltransferases (ORFs*_986*, *_999*, *_1005*, *_1008*, *_991* and *_866*) as well as the gene encoding a mannose-1-phosphate guanyltransferase (*cpsB*), belonging to that same class, were among the 544 genes repressed in the presence of apoplast fluid (S1 Table). Porin is involved in the exchange of nutrients over the outer membrane of Gram-negative bacteria but is also involved in pathogenesis [34]. It has been reported that the gene *cpsB* is related to extracellular polysaccharide biosynthesis and biofilm formation [35]. Extracellular polysaccharides play an important role in plant-bacteria interaction by promoting a firm and irreversible anchorage of the bacteria to the roots of plants [36, 37]. It has been verified that an EPS *G*. *diazotrophicus* strain PAL5 mutant (reduced production of exopolysaccharides) presented a deficient adhesion to the root surface of rice seedlings, whereas the complemented mutant reestablished this capacity similar to the wild type, indicating that EPS plays an important role in the initial steps of infection, leading to an efficient endophytic colonization [37].

Moreover, plants in general can recognize many bacterial Pathogen-associated molecular patterns (PAMPs). These PAMPs are also known as MAMPs (Microbe-Associated Molecular Pattern) because they refer to organisms that do not cause disease in plants such as endophytic diazotrophic bacteria. One example of MAMP peptide is the bacterial flagellin (FliC) that elicit a defense response in treated tomato cells and has the N-terminal 22 amino acids of the protein (flg22 peptide) that induces alkalinization of the extracellular media and production of reactive oxygen species (ROS) and ethylene [38]. Thereby the repression of FliC in the presence of apoplast fluid may be an attempt to escape plant defense responses. *Arabidopsis* plants with a defective defense system that prevent the activation of SAR (systemic acquired resistance) presented higher shoot and root growth when inoculated with *G*. *diazotrophicus* PAL5 strain, indicating that the bacteria may have activated the plant defense mechanisms [39]. Our hypothesis is that repression of these genes is part of the bacterial response to apoplast liquid compounds that may lead to modulation of LPS, EPS and peptidoglycan, therefore avoiding the elicitation of the defense response of the plant. Genes that participate in these pathways are part of biofilm synthesis that may be a virulence factor of several pathogens during the plant-bacteria interaction [40].

Genes encoding glycosyltransferases were also repressed (S1 Table). Glycosyltransferases transfer monosaccharides to acceptors with or without sugar, resulting in the formation of saccharide repeat units [41]. Once these units are polymerized, the formed polymer is exported from the cells and forms the EPS, LPS, CPS (capsular polysaccharide), peptoglycan, glycolipid and lipid glycosylation biomolecules [41]. Mutants of *X*. *citri* subsp. *citri* defective in the ability to produce glycosyltransferases showed reduced ability of both EPS and LPS biosynthesis as well as motility, biofilm formation and virulence in citrus plants [40]. Reduction of virulence was also verified in defective EPS-producing mutants of *X*. *campestris* pv. *campestris* [42]. These results suggest that glycosyltransferases may also play an important role in virulence and the repression of these genes may occur during the endophytic colonization of sugarcane tissues by strain Ppe8.

Since strain Ppe8 is an endophytic diazotrophic bacterium, it is possible that during the colonization process the expression of genes encoding the components of cell wall, membrane and envelope are repressed to bypass the plant defense responses and promote an efficient colonization. The results also suggest that *P*. *tropica* is able to control the synthesis of LPS in response to signals from the plant apoplast fluid compounds. LPSs are required for the colonization of maize by *H*. *seropedicae* as demonstrated for LPS deficient strains that showed a reduction of more than 90% in adhesion of the bacteria to the maize root surface [43]. The authors assumed that this event is the consequence of an inefficient adhesion and increased activation of plant defenses. Thus, our hypothesis is that after entering the plant, the bacterium represses the expression of these genes and consequently makes the interaction efficient and beneficial.

### Expression of genes involved in the cell motility and chemotaxis are affected in strain Ppe8 cultivated in presence of apoplast fluid

Bacterial colonization is dependent on motility and chemotaxis for attractive tags that may be compounds that activate bacterial-specific signaling pathways [44]. The *cheA*, *cheC*, *cheD*, *cheW*, *cheZ*, *cheY* and *cheR* genes (conserved group of a signal transduction system) encoding chemotaxis-related proteins and ORFs: _*1925*, _*3609*, _*5847*, _*6798* and _*7470* (known as MCPs or methyl-accepting proteins), a group of transmembrane chemoreceptors, were also repressed in strain Ppe8 in the presence of apoplast fluid (S1 Table). Genes encoding a central signal transduction pathway for chemotaxis are present in motile bacteria such as *Azospirillum* spp [45]. The group of genes mentioned above affects the production of exopolysaccharides and flocculation in the diazotrophic *Azospirillum brasilense* [46]. The signaling pathway of bacterial attraction by plant exudates involves a phosphorylation process that transduces chemo receptor signals to flagellar motors [47] and causes changes in the direction of motor rotation [48]. These receptors form complex associations with chemotactic proteins of the cytoplasm including histidine kinase, CheA and a CheW adapter protein. Upon receipt of the signal, CheA becomes autophosphorylated on a conserved histidine residue and connects to a CheY response regulator. The phosphorylated CheY controls the changes in the direction of the flagella. The activity of chemoreceptor signaling is modulated by antagonistic activity of a CheR methyltransferase and a CheB methylesterase. CheR adds methyl groups to specific glutamate residues in the C-terminal regions of receptors whereas CheB depends on phosphorylated CheA [48, 49]. This chemotactic signal transduction is conserved in both nearer and more phylogenetically distant bacterial species. Repression of flagella-related genes and chemotaxis indicates that apoplast fluid can reduce the motility of *P*. *tropica* strain PPe8. Genes related to chemotaxis are important mainly for the recognition of root exudates that activate some signaling pathways in bacteria and attract them to begin the plant tissue infection process [44]. It has already been demonstrated that some chemotaxis-related genes were repressed in *H*. *seropedicae* grown in the presence of naringenin [50]. A similar result was obtained when *Pseudomonas aeruginosa* strain PA01, a root colonizer of sugarbeet, was cultivated in the presence of root exudates of the *Beta vulgaris* L. (Celt variety), where the *cheY*, *cheA* and *pctA* genes were repressed [51]. Genes involved in chemotaxis were also repressed in *P*. *syringae* in the presence of bean apoplast fluid [32]. These results suggest that Ppe8 does not move because it finds the ideal niche in the liquid of the apoplast.

On the other hand, genes involved in assembling and rotating of the bacterial flagella such as *flgF*, *flhG*, *fliM*, *fliD*, *flhD*, *flhC*, *fliP1*, *fliD1*, *flgE*, *motA*, *motB*, *fliG*, *flgL* and *flgS* were repressed in the presence of the compounds of apoplast fluid (S1 Table). The transcriptomic analysis of *Bacillus subtilis* exposed for 2 hours to rice seedling extracts showed that genes like *fliL* and *flgK*, associated with motility, were repressed [52]. However, the expression of these genes evaluated by RT-qPCR confirmed their induction while genes involved in biofilm formation (*srfA* and *sinI*) remained induced for only the first 15 minutes. The authors concluded that the bacteria may have stopped their migration to the root until the harvest time (2 hours). This paralyzation or reduction of gene expression related to chemotaxis and movement may be a function of energy conservation that keep these genes at low levels [52]. The same may have happened with strain Ppe8 where the genes related to chemotaxis and movement were probably expressed faster and repressed later so that the bacterium could reduce its energy expenditure.

Our studies suggest that sugarcane apoplast fluid may affect the motility of *P*. *tropica* strain Ppe8. Considering that this study mimics what happens *in vivo*, the bacteria probably do not need to move towards the nutrient since it has already colonized the plant tissues and consequently the expression of such genes has already taken place when the bacteria were either in rhizoplane or at outer layer of roots. Therefore, our data indicate that active movement is less important when the bacterial population is well established within the plant. Our data corroborate those of [22], who found repressed motile-related genes in the transcriptome of *P*. *phytofirmans* grown inside potato plants. Another transcriptomic study involving *P*. *kururiensis* strain M130, cultivated in the presence of rice extract, showed that genes involved in flagella biosynthesis were mostly downregulated suggesting a mechanism that allows the bacteria to escape host plant defense responses [21].

### Catabolism of secondary metabolites and transport

Our analysis showed that *pcaH* and *pcaG* genes were induced in strain Ppe8 grown in sugarcane apoplast fluid and therefore may be involved in the degradation of compounds present in the apoplast fluid. The *pcaG* gene and others involved in the metabolism of phenolic compounds were also overexpressed in *Xanthomonas campestris* pv. *campestris* colonizing the xylem vessels of cabbage plants, indicating that they may be involved in the generation of precursors of the tricarboxylate cycle to provide energy [33]. Genes belonging to this metabolism were also overexpressed in *H*. *seropedicae* in the presence of naringenin [50]. Genes involved in the degradation of phenylacetic acid were also repressed in *Azoarcus* grown under nitrogen fixation conditions [53]. Our hypothesis is that Ppe8 has the ability to catabolize several types of aromatic compounds possibly present in the apoplast fluid to provide energy as a carbon source or to eliminate these toxic compounds.

### Signal transduction mechanisms

An interesting group that presented differentially expressed genes belongs to the signal transduction mechanism. Among the repressed genes in the strain Ppe8 grown in the presence of apoplast fluid are histidine kinases (ORFs _*7341*, _*3321*, _*5560*, and _*926*), responsive regulators (ORFs _*1924* and _*3322*), GGDEF, EAL, GAF or PAS/PAC domain proteins (ORFs _*4086*, _*1999*, _*4017*, _*4100*, _*6195*, _*1916* and _*5976*), among others (S1 Table). It is interesting to note that GGDEF or EAL domain proteins may be involved in cyclic di-GMP synthesis or hydrolysis, respectively, a global secondary messenger involved in signaling mechanisms for cell adhesion, motility, virulence, and morphogenesis [54–56]. The domains were repressed in strain Ppe8 in the presence of apoplast fluid indicating the repression of biofilm formation and motile-related genes. PAS/PAC domains are sensors that monitor changes in the environment of lightness, redox potential, oxygen or small molecules in the cytosol [57] and repression of these proteins may indicate that these variables were well controlled. Proteins with PAS/PAC domains may interact with hrp proteins that participate in the pathogenesis of *Xanthomonas axonopodis* pv. *citri* [58]. This may indicate that these domains also have a specific role in causing plant diseases and therefore the repression of these genes in strain Ppe8 would be a way to recognize sugarcane apoplast and consequently block the plant disease.

### Metabolism and transport of amino acids and carbohydrates

Large numbers of genes involved in metabolism and transport of amino acids were detected when the *Azoarcus* sp. BH72 strain was inoculated into rice. However, greater number of genes were expressed after one hour of exposure as compared to four-hour time, indicating that possibly the level of induction is a function of the degradation of compounds present in the medium [59]. A similar event may have taken place in the present work, where a larger number of genes were repressed after two hours of growth in presence of the apoplast fluid as compared to the defined medium.

Out of the 37 repressed genes in the presence of apoplast, 12 are ABC type sugar carriers. Genes encoding ABC type carriers were repressed in *H*. *seropedicae* strain Smr1 cultivated in the presence of naringenin [50]. It is important to note that *P*. *tropica* strain Ppe8 did not show differentially expressed genes related to sucrose metabolism, the main compound of the apoplast fluid [60]. All treatments began with 0.5% sucrose and for the gene induction there was a supplementation of 50 ml (0.5% sucrose—equivalent to 5 g/l) and 50 ml of apoplast fluid (approximately 11 g/l of sucrose—unpublished data) to growth both culture. Therefore, there was a high concentration of sucrose during this period although is it known that the apoplast fluid contains other sugar compounds.

In plants, arabinose and xylose are abundant sugars and cell wall components [61, 62]. Glucose and xylose are the main monosaccharides present in the cell wall of sugarcane, representing approximately 60% and 34%, respectively. Stem cells also contain fucose, galactose, arabinose, mannose and rhamnose in lower proportions [63]. This may mean, therefore, that the bacteria can repress genes related to the metabolism of these sugars in the presence of apoplast fluid and do not degrade the cell wall components surrounding the apoplast, thereby not becoming phytopathogenic. The phytopathogenic bacteria *B*. *glumae* induced the expression of these genes inside the rice tissues, because phytopathogens can metabolize these monosaccharides from the degradation of cell wall polymers during plant growth [64]. It is already known that arabinose and fucose also induce virulence genes in *Agrobacterium tumefaciens* [65].

The apoplast fluid of sugarcane varieties NCo310, RD 75–11 and PR 60–170, cultivated in Cuba, contains the amino acids serine, proline, alanine and aspartic acid in high proportion (about 60% of the total content) while glutamic acid, tyrosine, cysteine, methionine, glycine and lysine are also present but at lower concentrations and vary according to the variety and management (fertilization) [10]. Induction of genes involved in the metabolism of amino acids present in the apoplast fluid was confirmed, indicating that the bacteria are capable of metabolizing these amino acids (S1 Table). In addition, aromatic compounds from plants can be used as a carbon source by rhizospheric bacteria [66]. It is important to mention that two of the mentioned genes belong to nitrogen metabolism, histidine ammonia-lyase (*hutH*) and glutamate synthase (*glt1*). The *hutH* gene encodes an ammonia lyase that catalyzes the first step of degradation of histidine to produce urocanic acid while the *glt1* catalyzes the synthesis of glutamate by the reductive transfer of the amide group from glutamine to position 2 of 2-oxoglutarate, forming two molecules of glutamate. Both ammonia and urocanic acid are incorporated into glutamate metabolism, suggesting that this pathway is active when the bacteria are exposed to sugarcane apoplast fluid. Nitrate reductase and ammonia lyase genes were also induced in *P*. *syringae* grown in the presence of bean plant apoplast fluid [67].

ABC transporters represent a large active membrane transport superfamily of membrane proteins exhibiting a conserved domain (ATPase) that binds and hydrolyzes ATP, for the entry of various nutrients and the extrusion of drugs and residues of metabolites [68]. Genes encoding ABC-like transporters were repressed in *H*. *seropedicae* strain Smr1 in the presence of naringenin [50]. This repression could be an artifact of the bacteria to save energy since their metabolism is very active (induction of genes involved in translation class, ribosomal proteins and biogenesis). Another hypothesis is that these transporters were active at the begining and in order to reduce energy expenditure, the strain Ppe8 repressed the genes since the amino acids are already inside the cell and could be metabolized. The apoplast fluid repressed the expression of ORF*_7291* encoding a glutamine synthetase (GS) suggesting that ammonium, its main inhibitor, was probably in relatively greater amounts than in the defined medium. Glutamine synthetase also was repressed in *H*. *seropedicae* dependent on the amount of ammonium present in the culture medium [69].

## Conclusions

In this study, a comprehensive overview of *P*. *tropica* strain PPe8 transcriptome in the presence of the sugarcane variety RB867515 apoplast fluid was demonstrated. The results allowed inferences to be made about some aspects of the bacterial metabolism in presence and absence of sugarcane apoplast fluid. Expression of genes related to flagella biosynthesis, motor flagellum activity and chemotaxis were repressed by apoplast fluid, suggesting a negative effect on flagella synthesis and bacterial motility. The apoplast fluid also inhibited biosynthesis of exopolysaccharides. Expression of genes related to the catabolism of secondary metabolites and transport were induced because strain Ppe8 has the ability to catabolize several types of aromatic compounds possibly present in the apoplast fluid in order to provide energy as a carbon source or to eliminate toxic compounds.

This is a pioneering study on the interaction of *P*. *tropica* strain Ppe8 and sugarcane apoplast fluid and should support future (or ongoing) research in the field of Ppe8 functional genomics in response to compounds of different sugarcane varieties, hoping to maximize the plant response to inoculation with diazotrophic bacterial strains and consequently increasing the sugarcane yield. To our knowledge, this is the first report involving the expression of genes in compounds of sugarcane, a C4-energy plant colonized by many nitrogen-fixing endophytic bacteria, including the *P*. *tropica* strain Ppe8.

## Supporting information

S1 Table List of differentially expressed genes in *P. tropica* strain Ppe8 grown in presence of the appoplast fluid. (XLS)

## References

[1] Pechanova O, Hsu C-Y, Adams JP, Pechan T, Vandervelde L, Drnevich J, et al Apoplast proteome reveals that extracellular matrix contributes to multistress response in poplar. BMC genomics. 2010;11:674 10.1186/1471-2164-11-674 . 21114852 10.1186/1471-2164-11-674 PMC3091788

[2] Steudle E, Frensch J. Water transport in plants: Role of the apoplast. Plant and Soil. 1996;187:67–79.

[3] Dong Z, Canny MJ, McCully ME, Roboredo MR, Cabadilla CF, Ortega E, et al A nitrogen-fixing endophyte of sugarcane stems (a new role for the apoplast). Plant Physiology. 1994;105:1139–47. . 12232271 10.1104/pp.105.4.1139 PMC159442

[4] Fuentes-Ramírez L, Caballero-Mellado J, Sepúlveda J, Martínez-Romero E. Colonization of sugarcane by *Acetobacter diazotrophicus* is inhibited by high N-fertilization. FEMS Microbiology Ecology. 1999;29:117–28.

[5] Tejera N, Lluch C, Martìnez-Toledo MV, Gonzàlez-López J. Isolation and characterization of *Azotobacter* and *Azospirillum* strains from the sugarcane rhizosphere. Plant and Soil. 2005;270:223–32. 10.1007/s11104-004-1522-7

[6] Silva L, Miguens F, Olivares F. *Herbaspirillum seropedicae* and sugarcane endophytic interaction investigated by using high pressure freezing electron microscopy. Brazilian Journal of Microbiology. 2003;34:69–71.

[7] Dong Z, McCully ME, Canny MJ. Does *Acetobacter diazotrophicus* live and move in the xylem of sugarcane stems? Anatomical and physiological data. Annals of Botany. 1997;80:147–58.

[8] McCully ME. Niches for bacterial endophytes in crop plants: a plant biologist's view. Australian Journal of Plant Physiology. 2001;28(9):983–90.

[9] Asis Júnior C, Adachi K, Sugimoto A, Ujihara K, Terajima Y, Fukuhara S. Population of diazotrophic endophytes in stem apoplast solution of sugarcane and related grass species in Tanegashima, Japan. Microbes and Environments. 2003;18:133–7. 10.1264/jsme2.18.133

[10] Tejera N, Ortega E, Rodes R, Lluch C. Nitrogen compounds in the apoplastic sap of sugarcane stem: some implications in the association with endophytes. Journal of Plant Physiology. 2006;163:80–5. 10.1016/j.jplph.2005.03.010 . 16360806 10.1016/j.jplph.2005.03.010

[11] LPSN. Avaliable: http://www.bacterio.net/burkholderia.html; 2016 [cited 2016 05/01].

[12] Sawana A, Adeolu M, Gupta RS. Molecular signatures and phylogenomic analysis of the genus Burkholderia: proposal for division of this genus into the emended genus Burkholderia containing pathogenic organisms and a new genus Paraburkholderia gen. nov. harboring environmental species. Frontiers in Genetics. 2014;5:429 10.3389/fgene.2014.00429 . 25566316 10.3389/fgene.2014.00429 PMC4271702

[13] Oren A, Garrity GM. List of new names and new combinations previously effectively, but not validly, published. International Journal of Systematic and Evolutionary Microbiology. 2015;65:2017–25. 10.1099/ijs.0.000317 10.1099/ijsem.0.000632 28056215

[14] Caballero-Mellado J, Martínez-Aguilar L, Paredes-Valdez G, Estrada-De-Los Santos P. *Burkholderia unamae* sp. nov., an N2-fixing rhizospheric and endophytic species. International Journal of Systematic and Evolutionary Microbiology. 2004;54:1165–72. 10.1099/ijs.0.02951-0 . 15280286 10.1099/ijs.0.02951-0

[15] Reis VM, Estrada-de los Santos P, Tenorio-Salgado S, Vogel J, Stoffels M, Guyon S, et al *Burkholderia tropica* sp. nov., a novel nitrogen-fixing, plant-associated bacterium. International Journal of Systematic and Evolutionary Microbiology. 2004;54:2155–62. 10.1099/ijs.0.02879-0 . 15545451 10.1099/ijs.0.02879-0

[16] Perin L, Martínez-Aguilar L, Paredes-Valdez G, Baldani JI, Estrada-de Los Santos P, Reis VM, et al *Burkholderia silvatlantica* sp. nov., a diazotrophic bacterium associated with sugar cane and maize. International Journal of Systematic and Evolutionary Microbiology. 2006;56:1931–7. 10.1099/ijs.0.64362-0 . 16902033 10.1099/ijs.0.64362-0

[17] Schultz N, Morais RFD, Alves J, Baptista RB, Oliveira RP, Leite JM, et al Avaliação agronômica de variedades de cana-de-açúcar inoculadas com bactérias diazotróficas e adubadas com nitrogênio. Pesquisa Agropecuária Brasileira. 2012;47:261–8.

[18] Bernabeu PR, Pistorio M, Torres-Tejerizo G, Estrada-De-los Santos P, Galar ML, Boiardi JL, et al Colonization and plant growth-promotion of tomato by *Burkholderia tropica*. Scientia Horticulturae. 2015;191:113–20. 10.1016/j.scienta.2015.05.014

[19] Tenorio-Salgado S, Tinoco R, Vazquez-duhalt R, Caballero-Mellado J, Perez-Rueda E. Identification of volatile compounds produced by the bacterium *Burkholderia tropica* that inhibit the growth of fungal pathogens. Bioengineered. 2013;4:236–43. 10.4161/bioe.23808 23680857 10.4161/bioe.23808 PMC3728194

[20] Caballero-Mellado J, Onofre-Lemus J, Estrada-de Los Santos P, Martínez-Aguilar L. The tomato rhizosphere, an environment rich in nitrogen-fixing *Burkholderia* species with capabilities of interest for agriculture and bioremediation. Applied and environmental microbiology. 2007;73:5308–19. 10.1128/AEM.00324-07 . 17601817 10.1128/AEM.00324-07 PMC1950987

[21] Coutinho B, Licastro D, Mendonça-Previato L, Cámara M, Venturi V. Plant-Influenced Gene Expression in the Rice Endophyte *Burkholderia kururiensis* M130. Molecular Plant-Microbe Interactions. 2015;28:10–21. 10.1094/MPMI-07-14-0225-R 25494355 10.1094/MPMI-07-14-0225-R

[22] Sheibani-Tezerji R, Rattei T, Sessitsch A, Trognitz F. Transcriptome profiling of the endophyte *Burkholderia phytofirmans* PsJN indicates sensing of the plant environment and drought stress. mBio. 2015;6:1–11. 10.1128/mBio.00621-15.Invited 10.1128/mBio.00621-15 PMC4600099 26350963

[23] Baldani JI, Reis VM, Videira SS, Boddey LH, Baldani VLD. The art of isolating nitrogen-fixing bacteria from non-leguminous plants using N-free semi-solid media: a practical guide for microbiologists. Plant and Soil. 2014;384:413–31. 10.1007/s11104-014-2186-6

[24] Wu S, Zhu Z, Fu L, Niu B, Li W. WebMGA: a customizable web server for fast metagenomic sequence analysis. BMC Genomics. 2011;12:444 10.1186/1471-2164-12-444 21899761 10.1186/1471-2164-12-444 PMC3180703

[25] Rozen S, Skaletsky H. Primer3 on the WWW for general users and for biologist programmers. Bioinformatics Methods and Protocols. 1999;132:365–86. 10.1385/1-59259-192-2:365 10547847

[26] Silva PRA, Vidal MS, Soares CP, Polese V, Simões-Araújo JL, Baldani JI. Selection and evaluation of reference genes for RT-qPCR expression studies on *Burkholderia tropica* strain Ppe8, a sugarcane-associated diazotrophic bacterium grown with different carbon sources or sugarcane juice. Antonie van Leeuwenhoek. 2016;109(11):1493–502. 10.1007/s10482-016-0751-0 . 27535840 10.1007/s10482-016-0751-0

[27] Hellemans J, Mortier G, De Paepe A, Speleman F, Vandesompele J. qBase relative quantification framework and software for management and automated analysis of real-time quantitative PCR data. Genome Biology. 2007;8:R19 10.1186/gb-2007-8-2-r19 . 17291332 10.1186/gb-2007-8-2-r19 PMC1852402

[28] Tatusov RL, Koonin EV, Lipman DJ. A Genomic Perspective on Protein Families. Science. 1997;278:631–7. 10.1126/science.278.5338.631 . 9381173 10.1126/science.278.5338.631

[29] Russell MH. Characterization of the chemosensory abilities of the alphaproteobacterium Azospirillum brasilense. Dissertation presented for the Doctor of Philosophy Degree The University of Tennessee, Knoxville. 2012:1–282.

[30] Kanehisa M, Sato Y, Kawashima M, Furumichi M, Tanabe M. KEGG as a reference resource for gene and protein annotation. Nucleic Acids Research. 2016;44:D457–D62. 10.1093/nar/gkv1070 . 26476454 10.1093/nar/gkv1070 PMC4702792

[31] Jacobs JM, Milling A, Mitra RM, Hogan CS, Ailloud F, Prior P, et al Ralstonia solanacearum requires PopS, an ancient AvrE-family effector, for virulence and To overcome salicylic acid-mediated defenses during tomato pathogenesis. mBio. 2013;4:e00875–13. 10.1128/mBio.00875-13 . 24281716 10.1128/mBio.00875-13 PMC3870264

[32] Yu X, Lund SP, Scott Ra, Greenwald JW, Records aH, Nettleton D, et al Transcriptional responses of Pseudomonas syringae to growth in epiphytic versus apoplastic leaf sites. Proceedings of the National Academy of Sciences. 2013;110:E425–E34. 10.1073/pnas.1221892110 23319638 10.1073/pnas.1221892110 PMC3562829

[33] Bernonville TD, Noël LD, SanCristobal M, Danoun S, Becker A, Soreau P, et al Transcriptional reprogramming and phenotypical changes associated with growth of *Xanthomonas campestris* pv. *campestris* in cabbage xylem sap. FEMS Microbiology Ecology. 2014;89:527–41. 10.1111/1574-6941.12345 24784488 10.1111/1574-6941.12345

[34] Galdiero S, Falanga A, Cantisani M, Tarallo R, Elena Della Pepa M, D'Oriano V, et al Microbe-host interactions: structure and role of gram-negative bacterial porins. Current Protein and Peptide Science. 2012;13:843–54. 10.2174/138920312804871120 . 23305369 10.2174/138920312804871120 PMC3706956

[35] Lerner A, Castro-Sowinski S, Valverde A, Lerner H, Dror R, Okon Y, et al The *Azospirillum brasilense* Sp7 noeJ and noeL genes are involved in extracellular polysaccharide biosynthesis. Microbiology. 2009;155:4058–68. 10.1099/mic.0.031807-0 19762447 10.1099/mic.0.031807-0

[36] Steenhoudt O, Vanderleyden J. *Azospirillum*, a free-living nitrogen-fixing bacterium closely associated with grasses: genetic, biochemical and ecological aspects. FEMS Microbiology Reviews. 2000;24:487–506. 10.1111/j.1574-6976.2000.tb00552.x . 10978548 10.1111/j.1574-6976.2000.tb00552.x

[37] Meneses CHSG, Rouws LFM, Simoes-Araujo JL, Vidal MS, Baldani JI. Exopolysaccharide production is required for biofilm formation and plant colonization by the nitrogen-fixing endophyte *Gluconacetobacter diazotrophicus*. Molecular Plant-Microbe Interactions. 2011;24:1448–58. 10.1094/MPMI-05-11-0127 . 21809982 10.1094/MPMI-05-11-0127

[38] Mott GA, Middleton MA, Desveaux D, Guttman DS. Peptides and small molecules of the plant-pathogen apoplastic arena. Frontiers in Plant Science. 2014;5:1–12. 10.3389/fpls.2014.00677 . 25506352 10.3389/fpls.2014.00677 PMC4246658

[39] Rangel de Souza ALS, De Souza SA, De Oliveira MVV, Ferraz TM, Figueiredo FAMMA, Da Silva ND, et al Endophytic colonization of *Arabidopsis thaliana* by *Gluconacetobacter diazotrophicus* and its effect on plant growth promotion, plant physiology, and activation of plant defense. Plant and Soil. 2015;399:257–70. 10.1007/s11104-015-2672-5

[40] Danhorn T, Fuqua C. Biofilm formation by plant-associated bacteria. Annual Review of Microbiologyicrobiology. 2007;61:401–22. 10.1146/annurev.micro.61.080706.093316 . 17506679 10.1146/annurev.micro.61.080706.093316

[41] Li J, Wang N. The gpsX gene encoding a glycosyltransferase is important for polysaccharide production and required for full virulence in *Xanthomonas citri* subsp. citri. BMC Microbiology. 2012;12:31 10.1186/1471-2180-12-31 . 22404966 10.1186/1471-2180-12-31 PMC3364877

[42] Lu G-T, Ma Z-F, Hu J-R, Tang D-J, He Y-Q, Feng J-X, et al A novel locus involved in extracellular polysaccharide production and virulence of *Xanthomonas campestris* pathovar campestris. Microbiology. 2007;153:737–46. 10.1099/mic.0.2006/001388-0 . 17322194 10.1099/mic.0.2006/001388-0

[43] Balsanelli E, Serrato RV, de Baura Va, Sassaki G, Yates MG, Rigo LU, et al *Herbaspirillum seropedicae* rfbB and rfbC genes are required for maize colonization. Environmental Microbiology. 2010;12:2233–44. 10.1111/j.1462-2920.2010.02187.x . 21966916 10.1111/j.1462-2920.2010.02187.x

[44] Bashan Y, Holguin G, De-Bashan LE. Azospirillum-plant relationships: physiological, molecular, agricultural, and environmental advances (1997–2003). Canadian journal of microbiology. 2004;50:521–757. 10.1139/w04-035 . 15467782 10.1139/w04-035

[45] Sant'Anna FH, Almeida LGP, Cecagno R, Reolon La, Siqueira FM, Machado MRS, et al Genomic insights into the versatility of the plant growth-promoting bacterium *Azospirillum amazonense*. BMC Genomics. 2011;12:1–14. 10.1186/1471-2164-12-409 . 21838888 10.1186/1471-2164-12-409 PMC3169532

[46] Edwards AN, Siuti P, Bible AN, Alexandre G, Retterer ST, Doktycz MJ, et al Characterization of cell surface and extracellular matrix remodeling of *Azospirillum brasilense* chemotaxis-like 1 signal transduction pathway mutants by atomic force microscopy. FEMS Microbiology Letters. 2011;314:131–9. 10.1111/j.1574-6968.2010.02156.x . 21105907 10.1111/j.1574-6968.2010.02156.x

[47] Celani A, Vergassola M. Bacterial strategies for chemotaxis response. Proceedings of the National Academy of Sciences of the United States of America. 2010;107:1391–6. 10.1073/pnas.0909673107 . 20080704 10.1073/pnas.0909673107 PMC2824349

[48] Wadhams GH, Armitage JP. Making sense of it all: bacterial chemotaxis. Nature Reviews Molecular Cell Biology. 2004;5:1024–37. 10.1038/nrm1524 . 15573139 10.1038/nrm1524

[49] Sourjik V. Receptor clustering and signal processing in *E*. *coli* chemotaxis. Trends in Microbiology. 2004;12:569–76. 10.1016/j.tim.2004.10.003 . 15539117 10.1016/j.tim.2004.10.003

[50] Tadra-Sfeir MZ, Faoro H, Camilios-Neto D, Brusamarello-Santos L, Balsanelli E, Weiss V, et al Genome wide transcriptional profiling of *Herbaspirillum seropedicae* SmR1 grown in the presence of naringenin. Frontiers in Microbiology. 2015;6:1–8. 10.3389/fmicb.2015.00001 26052319 10.3389/fmicb.2015.00491 PMC4440368

[51] Mark GL, Dow JM, Kiely PD, Higgins H, Haynes J, Baysse C, et al Transcriptome profiling of bacterial responses to root exudates identifies genes involved in microbe-plant interactions. Proceedings of the National Academy of Sciences of the United States of America. 2005;102:17454–9. 10.1073/pnas.0506407102 . 16301542 10.1073/pnas.0506407102 PMC1297666

[52] Xie S, Wu H, Chen L, Zang H, Xie Y, Gao X. Transcriptome profiling of *Bacillus subtilis* OKB105 in response to rice seedlings. BMC Microbiology. 2015;15:21 10.1186/s12866-015-0353-4 . 25651892 10.1186/s12866-015-0353-4 PMC4326333

[53] Sarkar A, Reinhold-Hurek B. Transcriptional profiling of nitrogen fixation and the role of NifA in the diazotrophic endophyte *Azoarcus* sp. strain BH72. PloS One. 2014;9:e86527 10.1371/journal.pone.0086527 . 24516534 10.1371/journal.pone.0086527 PMC3916325

[54] Simm R, Morr M, Kader A, Nimtz M, Römling U. GGDEF and EAL domains inversely regulate cyclic di-GMP levels and transition from sessility to motility. Molecular Microbiology. 2004;53:1123–34. 10.1111/j.1365-2958.2004.04206.x . 15306016 10.1111/j.1365-2958.2004.04206.x

[55] Römling U, Gomelsky M, Galperin MY. C-di-GMP: the dawning of a novel bacterial signalling system. Molecular Microbiology. 2005;57:629–39. 10.1111/j.1365-2958.2005.04697.x . 16045609 10.1111/j.1365-2958.2005.04697.x

[56] Boyd CD, O'Toole Ga. Second messenger regulation of biofilm formation: breakthroughs in understanding c-di-GMP effector systems. Annual Review of Cell and Developmental Biology. 2012;28:439–62. 10.1146/annurev-cellbio-101011-155705 23057745 10.1146/annurev-cellbio-101011-155705 PMC4936406

[57] Taylor B, Zhulin I. PAS domains: internal sensors of oxygen, redox potential, and light. Microbiology and Molecular Biology Reviews. 1999;63:479–506. 10357859 10.1128/mmbr.63.2.479-506.1999 PMC98974

[58] Alegria MC, Docena C, Khater L, Ramos CHI, Silva ACR, Farah CS. New protein-protein interactions identified for the regulatory and structural components and substrates of the type III secretion system of the phytopathogen *Xanthomonas axonopodis* pathovar *citri*. Journal of Bacteriology. 2004;186:6186–97. 10.1128/JB.186.18.6186-6197.2004 15342589 10.1128/JB.186.18.6186-6197.2004 PMC515140

[59] Shidore T, Dinse T, Öhrlein J, Becker A, Reinhold-Hurek B. Transcriptomic analysis of responses to exudates reveal genes required for rhizosphere competence of the endophyte *Azoarcus* sp. strain BH72. Environmental Microbiology. 2012;14:2775–87. 10.1111/j.1462-2920.2012.02777.x . 22616609 10.1111/j.1462-2920.2012.02777.x

[60] Asis Júnior C, Katsuki A, Akao S. N2 fixation in sugarcane and population of N2-fixing endophytes in stem apoplast solution. Philippine Journal of Crop Science. 2004;29:45–58.

[61] Burget EG, Verma R, Mølhøj M, Reiter W-d. The biosynthesis of L-arabinose in plants: molecular cloning and characterization of a golgi-localized UDP-D-xylose 4-epimerase encoded by the MUR4 gene of Arabidopsis. The Plant Cell. 2003;15:523–31. 10.1105/tpc.008425 12566589 10.1105/tpc.008425 PMC141218

[62] Desai TA, Rao CV. Regulation of arabinose and xylose metabolism in *Escherichia coli*. Applied and Environmental Microbiology. 2009;76:1524–32. 10.1128/AEM.01970-09 20023096 10.1128/AEM.01970-09 PMC2832368

[63] de Souza AP, Leite DCC, Pattathil S, Hahn MG, Buckeridge MS. Composition and structure of sugarcane cell wall polysaccharides: implications for second-generation bioethanol production. BioEnergy Research. 2012;6:564–79. 10.1007/s12155-012-9268-1

[64] Kim S, Park J, Lee J, Shin D, Park D-S, Lim J-S, et al Understanding pathogenic *Burkholderia glumae* metabolic and signaling pathways within rice tissues through in vivo transcriptome analyses. Gene. 2014;547:77–85. 10.1016/j.gene.2014.06.029 . 24949534 10.1016/j.gene.2014.06.029

[65] Cangelosi GA, Ankenbauer RG, Nestert EW. Sugars induce the *Agrobacterium virulence* genes through a periplasmic binding protein and a transmembrane signal protein. Proceedings of the National Academy of Sciences USA. 1990;87:6708–12. 10.1073/pnas.87.17.6708 PMC54606 2118656

[66] Ramachandran VK, East AK, Karunakaran R, Downie JA, Poole PS. Adaptation of *Rhizobium leguminosarum* to pea, alfalfa and sugar beet rhizospheres investigated by comparative transcriptomics. Genome Biology. 2011;12:R106 10.1186/gb-2011-12-10-r106 . 22018401 10.1186/gb-2011-12-10-r106 PMC3333776

[67] Hernández-Morales A, De la Torre-Zavala S, Ibarra-Laclette E, Hernández-Flores JL, Jofre-Garfias AE, Martínez-Antonio A, et al Transcriptional profile of *Pseudomonas syringae* pv. *phaseolicola* NPS3121 in response to tissue extracts from a susceptible *Phaseolus vulgaris* L. cultivar. BMC Microbiology. 2009;9:257 10.1186/1471-2180-9-257 . 20003402 10.1186/1471-2180-9-257 PMC2803797

[68] Nicolás MF, Barcellos FG, Hess PN, Hungria M. ABC transporters in *Mycoplasma hyopneumoniae* and *Mycoplasma synoviae*: Insights into evolution and pathogenicity. Genetics and Molecular Biology. 2007;30:202–11.

[69] Cordeiro F, Tadra-Sfeir M, Huergo L, Pedrosa F, Monteiro R, Souza E. Proteomic analysis of *Herbaspirillum seropedicae* cultivated in the presence of sugar cane extract. Journal of Proteome Research. 2013;12:1142–50. 10.1021/pr300746j . 23331092 10.1021/pr300746j

